# Breaking Down a Rhythm: Dissecting the Mechanisms Underlying Task-Related Neural Oscillations

**DOI:** 10.3389/fncir.2022.846905

**Published:** 2022-03-04

**Authors:** Inés Ibarra-Lecue, Saskia Haegens, Alexander Z. Harris

**Affiliations:** ^1^Department of Psychiatry, College of Physicians and Surgeons, Columbia University, New York, NY, United States; ^2^New York State Psychiatric Institute, New York, NY, United States; ^3^Donders Centre for Cognitive Neuroimaging, Donders Institute for Brain, Cognition and Behaviour, Radboud University, Nijmegen, Netherlands

**Keywords:** electrophysiology, optogenetic stimulation, optogenetic inhibition, alpha oscillations, beta oscillations, theta oscillations, delta oscillations, gamma oscillations

## Abstract

A century worth of research has linked multiple cognitive, perceptual and behavioral states to various brain oscillations. However, the mechanistic roles and circuit underpinnings of these oscillations remain an area of active study. In this review, we argue that the advent of optogenetic and related systems neuroscience techniques has shifted the field from correlational to causal observations regarding the role of oscillations in brain function. As a result, studying brain rhythms associated with behavior can provide insight at different levels, such as decoding task-relevant information, mapping relevant circuits or determining key proteins involved in rhythmicity. We summarize recent advances in this field, highlighting the methods that are being used for this purpose, and discussing their relative strengths and limitations. We conclude with promising future approaches that will help unravel the functional role of brain rhythms in orchestrating the repertoire of complex behavior.

## Introduction

### History

In the early 20th century, Berger (1929) discovered that the electrical activity of neurons can be recorded from electrodes placed on the scalp. These electrical recordings, known as electroencephalography (EEG), reflect the summed synaptic activity of large neuronal populations (Buzsaki et al., 2012) and reveal that brain activity is highly rhythmic. Berger (1929) reported that activity at approximately 10 Hz – the so-called alpha rhythm – is strongest during eye closure and rest, and reduced by visual stimulation (Mazaheri et al., 2014). Subsequent research using not only EEG, but also other non-invasive techniques such as magnetoencephalography (MEG), and invasive recordings of the local field potential (LFP), has associated different behavioral and cognitive processes with particular brain regions and oscillatory dynamics. For example, hippocampal theta oscillations (4–8 Hz) have been linked with episodic memory and navigation (McNaughton et al., 2006; Buzsáki and Moser, 2013), alpha oscillations (8–14 Hz) with sensory processing and attention (Haegens et al., 2011a; Mazaheri et al., 2014; Samaha et al., 2020; Zhou et al., 2021), cortical beta (15–30 Hz) with working memory and perceptual decision making (Haegens et al., 2011b; Herding et al., 2016; Spitzer and Haegens, 2017), and gamma (30–90 Hz) in a variety of different brain regions with sensory processing, cognition, memory, and attention (Cardin et al., 2009; Lundqvist et al., 2018; Kanta et al., 2019; Zielinski et al., 2019). Recently, the field has moved away from simply correlating oscillations with behavior. Instead, new theoretical frameworks propose that different rhythms reflect distinct lower-level functions (such as modulating gain or facilitating synchrony) that are flexibly employed to change specific neuronal population dynamics, based on the information processing required for the behavioral context. Defining these lower-level functions involves an ongoing challenge that represents one main approach to studying oscillations in behavior. Another fruitful approach involves using oscillations as a window into the dynamic activity of neuronal circuits that mediate specific behaviors, by determining the contribution of neural subpopulations to the oscillations that emerge during behavior. We will summarize these two approaches which, while having different scientific goals, can unravel complementary questions about brain processing.

### Toward Understanding the Fundamental Role of Oscillations

Neural oscillations emerge from synchronized synaptic activity, resulting in periodic collective shifts between higher and lower intracellular voltage or excitability states (Bishop, 1932). Neural oscillations have been proposed to facilitate neural communication (Fries, 2015; Harris and Gordon, 2015), either by synchronizing distant brain regions to align inter-regional information transfer (Varela et al., 2001; Jones and Wilson, 2005a; Adhikari et al., 2010), by facilitating the perception of disparate features as a unified object (Gray, 1999; Engel and Singer, 2001), or by organizing the neural activity of local ensembles (O’keefe and Recce, 1993; Csicsvari et al., 2000; Fries, 2001; Dejean et al., 2016). Ongoing efforts aim to uncover the underlying principles by which different rhythms support brain operation.

**FIGURE 1:**
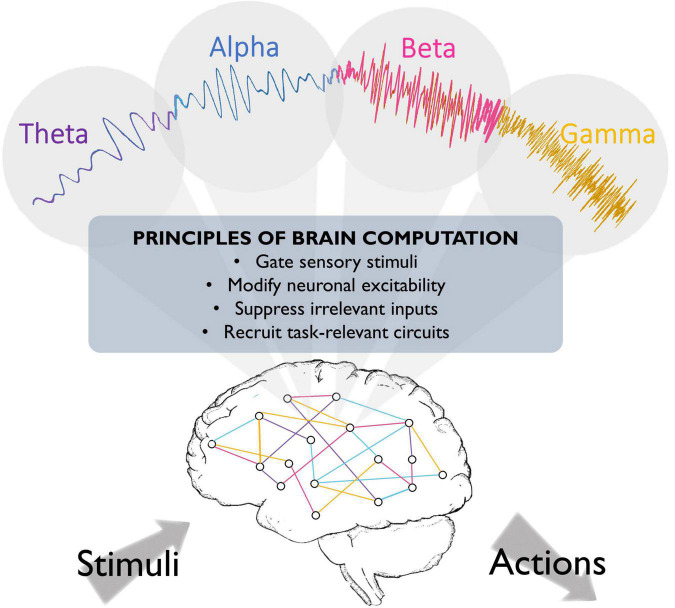
Fundamental roles that oscillations may play in brain function. Brain oscillations have been implicated in crucial aspects of neural circuit activity, such as stimulus perception. They have also been suggested to play a role in top-down processing, such that different oscillations might emerge to engage task-relevant circuits and silence irrelevant information, thereby optimizing brain processing and behavioral performance.

A recent proposal suggests that different rhythms cooperate to change the dynamics of neuronal populations, thereby preparing the system for the ongoing task (see Figure 1). In this scheme, oscillations in different frequency bands may serve distinct functions. Slow (<8 Hz) oscillations seem to provide the temporal framework for sensory selection and encoding (Lakatos et al., 2008; Schroeder and Lakatos, 2009). Slow coherent oscillations between networks are thought to enhance effective communication between regions, as coherence increases in behaviorally relevant periods and predicts learning (Decoteau et al., 2007). During sleep, different low frequencies dominate during different phases (theta oscillations in the rapid-eye-movement (REM) phase, and slow waves in the delta (1–4 Hz) range in the non-REM phase), and seem to play complementary roles in memory consolidation (Diekelmann and Born, 2010; Buzsáki and Moser, 2013; Jacobs, 2014). Moreover, hippocampal theta phase coding, known as phase precession, has been suggested to organize the spike timing of individual neurons both locally and in distant brain regions (O’keefe and Recce, 1993; Jones and Wilson, 2005a; Van Der Meer and Redish, 2011). In addition, slow oscillations organize faster rhythms, reflected in phase-amplitude coupling, where each cycle of the slower rhythm provides a window-of-opportunity for faster beta/gamma oscillations. These rhythmic interactions have been implicated in spatial working memory performance and memory processing during sleep (Jones and Wilson, 2005a,b; Decoteau et al., 2007; Axmacher et al., 2010; Arnal et al., 2015; Antony et al., 2019). Alpha oscillations have been proposed to reflect suppression of irrelevant inputs (Klimesch et al., 2007), as pre-stimulus alpha power inversely correlates with firing rates and lower alpha in task-relevant regions predicts enhanced sensory perception (Thut, 2006; Haegens et al., 2011c; Van Kerkoerle et al., 2014), while concurrently, alpha power increases in task-irrelevant regions (Klimesch et al., 1999; Jensen et al., 2002). We recently suggested that beta oscillations reflect reactivation of neural ensembles when previously encoded information is required for subsequent processing steps such as decision making (Spitzer and Haegens, 2017). This model is in agreement with evidence of content-specific beta power modulations in multiple areas including the prefrontal cortex, which reflect task-relevant stimulus features maintained in working memory, and predict subsequent decision outcomes (Spitzer and Blankenburg, 2012; Haegens et al., 2017). Finally, gamma oscillations are suggested to reflect neural excitation, circuit engagement, and consequent amplification of input, given that sensory stimulation evokes gamma rhythms in sensory areas, and attending to different sensory stimuli increases evoked gamma power (Tiitinen et al., 1993; Fries, 2001; Bentley et al., 2016; Kim et al., 2016), predicts faster visual perception (Womelsdorf et al., 2006) and successful memory formation (Sederberg et al., 2006), and usually correlates with increased neural firing rates (Ray et al., 2008; Manning et al., 2009). This rhythm may also support precise inter-area communication, as gamma-synchronization increases during visual processing and cognitive tasks, and may support enhanced cognitive flexibility (Fries, 2009; Igarashi et al., 2014; Yamamoto et al., 2014; Cho et al., 2020; Fernández-Ruiz et al., 2021).

Thus, an important goal of studying oscillations is to understand their fundamental functional roles. Indeed, one of the main challenges of this endeavor is to identify the crucial parameters that define (or the level at which one could define) these roles. To do so, collecting evidence from different behavioral paradigms, brain regions, and species is crucial. In this regard, interesting evidence indicates that slower hippocampal oscillations organize faster rhythms and PFC cell assemblies, and predict memory consolidation across species (Louie and Wilson, 2001; Benchenane et al., 2010; Boyce et al., 2016; Schreiner et al., 2021). However, functionally equivalent oscillations can cross the traditional frequency boundaries, depending on the species, strain, or even individuals (Haegens et al., 2014; Tort et al., 2018a; Halgren et al., 2019). For example, while rodent hippocampal theta rhythms represent one of the most well-understood oscillations (Hasselmo, 2005), human hippocampal “theta” rhythms may actually fall in the delta range (Watrous et al., 2013; Jacobs, 2014). In mice, we recently found that the same stressor induced nucleus accumbens oscillations in the delta or the theta range depending on the strain, suggesting genetic differences play a role in frequency boundaries (Lowes et al., 2021). Similarly, some individuals, have beta oscillations that function the way alpha rhythms do in other individuals (Haegens et al., 2014). These examples argue that strictly adhering to traditional frequency-based nomenclatures to describe brain rhythms may hinder efforts to address their functional role. Instead, rhythms arising from the same circuit and neuronal subpopulations may play a similar functional role across species while differing in their frequency, as has been suggested for hippocampal theta (Jacobs, 2014). Thus, we suggest that, when possible, studies should refer to oscillations by the circuit and conditions that elicit them, rather than simply by strict frequency ranges.

Developing this circuit-based taxonomy of oscillations requires better understanding of the underlying neural activity, but may help resolve seemingly contradictory functional consequences of oscillations (Shin et al., 2017; Lundqvist et al., 2018). For example, beta oscillations have been proposed to serve as an inhibitory mechanism in the sensorimotor system (Donner et al., 2009; Pogosyan et al., 2009). However, during working memory, beta oscillations in other regions including prefrontal cortex seem to reflect formation and endogenous reactivation of specific neuronal ensembles for subsequent processing, with conflicting evidence about whether it provides local inhibition or excitation (Haegens et al., 2011b; Spitzer and Haegens, 2017; Miller et al., 2018). Determining the neural populations that produce these respective beta oscillations may explain this apparent discrepancy. Prior efforts to dissect simultaneous rhythms using pharmacological interventions have helped unravel decades-lasting debates, such as the existence of at least two different hippocampal oscillations that emerge from different cell types during anesthesia and active exploration (Kramis et al., 1975), and are still used for dissecting the functional role of different hippocampal theta rhythms (Park et al., 2021).

These results highlight the utility of invasive, intracortical recordings, which provide better spatial resolution of the rhythmic activity (since they are less confounded by volume conduction) as well as measures of individual neuronal activity. Evaluating action potentials can reveal local influences of oscillations on the timing of neuronal spikes, which provides further evidence that the pertinent oscillation is not simply volume conducted (Shin et al., 2017; Lundqvist et al., 2018). Furthermore, this analysis can help address open questions such as the impact of beta oscillations on neuronal activity (Spitzer and Haegens, 2017; Lundqvist et al., 2018), or how the actual information is encoded (Romo et al., 1999; Barak et al., 2010; Vergara et al., 2016). Analysis of the relationship between firing rates and oscillations extended to long-range circuits can also provide strong evidence regarding the source of oscillatory activity, by identifying the neuronal populations that spontaneously fire at specific frequencies, thereby driving synchrony and generating oscillatory activity under different circumstances. Some examples of successfully exploiting this relationship include the discovery of a subset of thalamocortical neurons with intrinsic burst firing that are implicated in the generation of alpha rhythms (Lorincz et al., 2008), the role of GABA neurons within the medial septum in generating hippocampal theta (Hangya et al., 2009), and of inhibitory interneurons in gamma oscillations (Whittington et al., 1995; Cardin et al., 2009; Sohal et al., 2009; Kim et al., 2016).

Ultimately, causal interventions manipulating neural populations can definitively demonstrate the neural origins of specific oscillations. This approach has the additional advantage of providing insight into the functional relevance of oscillations. As we will discuss below, such causal interventions are ushering in an exciting new era of neural oscillations research. These experiments require rodents – species that allows invasive experiments – and rest on two assumptions: (1) that the same behavioral tasks require similar neuronal circuits across different species; and (2) that involved neuronal circuits show similar oscillatory activity between species, when engaged in similar tasks. The first assumption has some long-standing evidence, such as the studies of the primary visual cortex and its role in visual processing across different species, pioneered in the early 19th century by anatomist Bartolomeo Panizza (Mazzarello and Sala, 1993). More recent studies include demonstrations that V1 neuronal activity is similarly modulated in visual perception tasks in humans (Tootell et al., 1998), rodents (Cone et al., 2020), and cats (Hua et al., 2010). However, the second assumption remains more controversial, as evidenced by the previously mentioned differences between hippocampal rhythms in mice and humans (Lisman and Idiart, 1995; Lisman and Jensen, 2013; Jacobs, 2014; Boyce et al., 2016; Vass et al., 2016). Given the inter-species [and inter-individual (Haegens et al., 2014)] variation in frequency discussed earlier, we propose that oscillation studies should be grounded in which behavioral conditions, neural circuits, neural subpopulations, or even ion channels (Kalmbach et al., 2018; Stagkourakis et al., 2018), elicit the oscillation. To do so, it will be critical to conduct carefully designed parallel human-murine studies which use equivalent tasks (Cavanagh et al., 2021), identify the human EEG source with sophisticated source localization algorithms (Michel and Brunet, 2019; Seeber et al., 2019) and/or functional imaging, and confirm similar neural population involvement with pharmacology when possible.

In summary, disentangling exactly how and where these rhythms are generated in the brain may provide more definitive evidence of what oscillations mean for brain processing, as well as how different circuits encode and route the information that is needed for specific behaviors and, in general, for an efficient interaction with the world.

### Rhythms as a Window to Understand the Neuronal Circuits Underlying Behavior

A complementary approach to studying brain rhythms views the oscillations that accompany behavior as a tool that yields insight into the underlying neurons and circuits. The main goal of this approach is to understand the specific neuronal circuits and cellular populations that are necessary for different behaviors. As a result, state-induced oscillatory activity helps track activated areas and synchronized brain regions (Hultman et al., 2018; Schneider et al., 2021), to evaluate which neuronal populations drive the activity, and their impact on behavior (Harris and Gordon, 2015). Unlike the approach described above, these investigations are not driven by specific hypotheses about general functional roles of specific rhythms. Instead, what is key for this approach is tightly linking the oscillation to the specific behavior on the one hand, and to underlying neurons and circuits, on the other. Once there is evidence that oscillatory activity is related to a particular behavior, a key aspect of this approach is to evaluate the effect of the ongoing oscillatory activity in the firing rates of local or distant areas, to find the neural populations that are synchronized, i.e., phase locked, and/or modulated by the oscillations. This analysis makes it possible to identify the key circuit nodes within a larger network that underlie a given behavior as well as to determine which neurons mediate the neural activity that supports that behavior.

To illustrate this approach, recent studies using intracortical recordings have observed that low-frequency (2–7 Hz) oscillations emerge during stressful and anxiety-like conditions in many limbic areas such as the ventral tegmental area (VTA), nucleus accumbens (NAc), amygdala and prelimbic cortex (Kumar et al., 2014; Karalis et al., 2016; Lowes et al., 2021). These brain areas play crucial roles in mediating such disparate consequences of stress as decreased reward seeking and fear-induced freezing. Investigating the circuit basis of these rhythms in different behavioral contexts reveals that the low-frequency rhythm that accompanies stress-induced deficits in reward seeking reflects VTA to NAc circuit activity (Lowes et al., 2021). By contrast, freezing behavior co-occurs with a synchronization of prelimbic cortex and amygdala (Lesting et al., 2011; Likhtik et al., 2014; Stujenske et al., 2014; Karalis et al., 2016). Interestingly, this cortico-amygdala synchrony may arise from respiration-induced activity in the olfactory bulb (Ito et al., 2014; Tort et al., 2018a; Bagur et al., 2021), while the VTA-NAc does not correlate with respiration (Lowes et al., 2021). These examples illustrate how oscillations can help identify the unique circuits relevant to particular behaviors. As can be seen from these examples, the same frequency range may reflect different circuit activity with different behavioral consequences. Indeed, different researchers have published competing interpretations of which circuits produce this frequency range activity (Roy et al., 2017; Tort et al., 2018b). Fortunately, spatially precise manipulations allow us to dissect the different circuits that yield similar frequency oscillations and determine different aspects of behavior.

Box 1. Optogenetics refers to a combination of biological techniques that makes it possible to use light to control cell physiology (Fenno et al., 2011). Using genetic engineering methods, transmembrane light-sensitive proteins (known as opsins) are artificially expressed in the cells of interest. Opsins covalently bind a specific molecule – chromophore – which, upon photon absorption, transiently changes its conformation and allows ions to pass through the plasma membrane, thereby regulating the electrical activity of the cell. This technique can be applied to nearly every cell type, either by a viral delivery of the opsin gene, or by generating transgenic lines that express the opsin with the promoter of a gene of interest. The major role that electrical activity plays in generating action potentials has made optogenetics especially applicable for neuronal activity manipulation. Optogenetics pioneer Karl Deisseroth and colleagues first generated light-evoked neuronal spikes *ex vivo* in 2005, by (1) modifying a lentivirus to contain a opsin gene, originally from unicellular algae, (2) infecting a rat hippocampal cell culture with the virus, and (3) inducing rapid depolarizing currents that trigger action potentials with blue light pulses (Boyden et al., 2005). The first successful *in vivo* study with a mouse line expressing opsins in the central nervous system came a few years later, showing that this technique can reliably generate firing rates as high as 40 Hz in individual neurons of the olfactory bulb (Arenkiel et al., 2007). Since then, this technique has become very powerful, as it can be implemented in awake, behaving rodents, and combined with electrophysiology probes or neurotransmitter sensors (Cardin et al., 2010; Kim et al., 2017). Opsins modulate neural activity with high temporal and spatial resolution, which ultimately depend on the opsin type, light source, and tissue characteristics. See Boyden (2011) for an in-depth historical overview on the field, and (Yizhar et al., 2011) for a comprehensive technical review of this rapidly developing field and its main challenges. Briefly, optogenetic stimulation involves the expression of an ion channel that in response to light opens and lets multiple cations diffuse into the cytosol, evoking neuronal depolarization and triggering action potentials. The most used excitatory opsin is the channelrhodopsin, which responds to blue light. Currently, there are multiple excitatory opsins that display different photocurrent amplitudes and kinetics and can be activated with different light wavelengths. For example, stable step-function or bi-stable opsins have an increased open-state lifetime of several minutes, allowing long periods of a higher-rate spontaneous spiking pattern of defined neurons (Yizhar et al., 2011). Other examples are Chronos, with faster kinetic parameters; and Chrimson, which activation spectrum is substantially red-shifted (Klapoetke et al., 2014). Optogenetic inhibition, in contrast, is used to hyperpolarize the neurons and silence them with light. This hyperpolarization is attained through the expression of ion pumps that respond to green-yellow light, either pumping chloride anions inside the cell (halorhodopsins) or pumping protons out (archaerhodopsins) (Zhang et al., 2007). Similarly to the stimulation approach, these opsins have been engineered to achieve enhanced performance, such as eNpHR, which has increased photocurrent amplitudes (Gradinaru et al., 2008). Crucially, the expression of the opsins can be restricted to different cell populations by using gene-targeting technology such as specialized mouse lines. These mice lines have been engineered to express a gene-editing enzyme (Cre recombinase) under cell-specific promoters [such as glutamate transporter or choline acetyltransferase (Borgius et al., 2010; Rossi et al., 2011)]. This enzyme binds specific gene sequences, called loxP sites, and rearranges any DNA fragments that are flanked between two loxP sites. This technology allows a viral vector to deliver active opsins selectively to the cells with the promoter of interest, allowing regional and cell subtype specific manipulation of neuronal activity.

## Using Causal Interventions to Interrogate the Role of Neural Oscillations

While current efforts provide a better insight into how different oscillations may function together to tune different task-relevant brain circuits underlying the complex computations that guide behavior, several outstanding questions remain. First, are these oscillations necessary for the proposed neural function? Second, does the oscillatory activity cause the behavioral effect? Third, which neural elements underlie an oscillation in a given behavioral condition? As different oscillations seem to have opposite effects on neuronal activity, suggesting that they might differ in their underlying neuronal circuits, it is crucial to identify their neurophysiological underpinnings. In the following section, we will highlight new approaches to tackle these questions using optogenetics (see Box 1), which makes it possible to manipulate specific local oscillatory dynamics with high temporal and spatial precision (Boyden et al., 2005; Adamantidis et al., 2007; Zhang et al., 2007). We aim to provide a roadmap for evaluating the theories regarding functional/mechanistic roles of oscillations in awake-behaving animal models.

**Box 2. Illustrative example of an experimental design and data analysis for dissecting the circuit underlying a behaviorally relevant oscillation.**

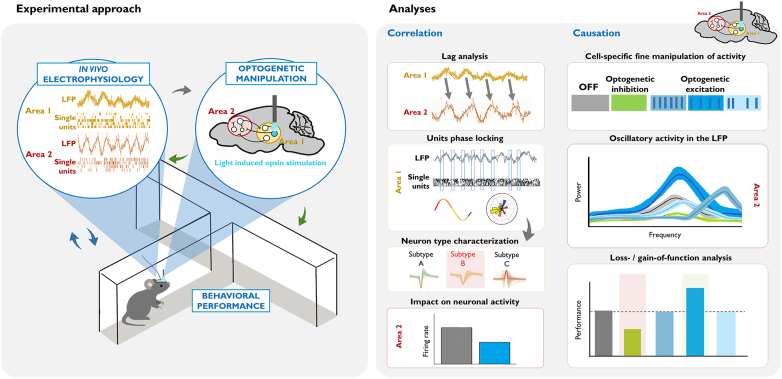

Left: Combining *in vivo* invasive electrophysiological recordings in awake-behaving rodents with cell-type specific optogenetic manipulation. Oscillatory activity (in the LFP or single unit recordings) is correlated with particular behaviors or states (blue arrows). The virus, opsin type, mouse model, area, and pattern of the optogenetic manipulation are chosen based on this neural activity and the goals of the study (gray arrow). Crucially, the consequences of these causal manipulations of neuronal activity are evaluated, in terms of electrophysiological measurements in the area(s) of interest, and their behavioral correlates (green arrows).

Right: Summary of commonly used analyses for dissecting the circuits and neuronal populations underlying a task-relevant oscillation. *Correlation studies:* In this example, lag analysis of the LFP of two areas shows that past oscillatory activity in Area 1 predicts the future oscillatory activity of Area 2 (see the shift in the phase, and that the frequency of the LFP signal is similar). Phase locking analysis of Area 1 activity demonstrates that there is entrainment of local single units, such that they fire at a specific phase of the oscillation. Subsequent analysis of different action potential parameters within the phase-locked neurons (gray arrow) can help identify a putative neuronal subtype that drives the oscillation. Measuring net firing rates in Area 2 can help identify whether the oscillation reflects global excitatory or inhibitory input. *Causation studies:* Once there is evidence suggesting that a specific cell subtype causes the oscillation, different optogenetic approaches can be used in order to demonstrate the necessity and/or sufficiency of that cell type for the oscillation, and in turn, for the particular behavior. In this example, optogenetic inhibition of the cell inhibits the naturally occurring oscillatory activity in Area 2, and decreases behavioral performance (see green vs. gray data in the graphics). Optogenetic excitation allows for precise control of the neuronal activity. Here, we illustrate three patterns of stimulation: higher frequency rhythmic, lower frequency rhythmic, and arrhythmic. In this example, lower frequency rhythmic is the only pattern that produces an oscillation of a similar frequency (see the similar position of the bright blue and gray peaks at the frequency axis), and evokes a behavioral output.

### Addressing Oscillations With Optogenetics

**FIGURE 2:**
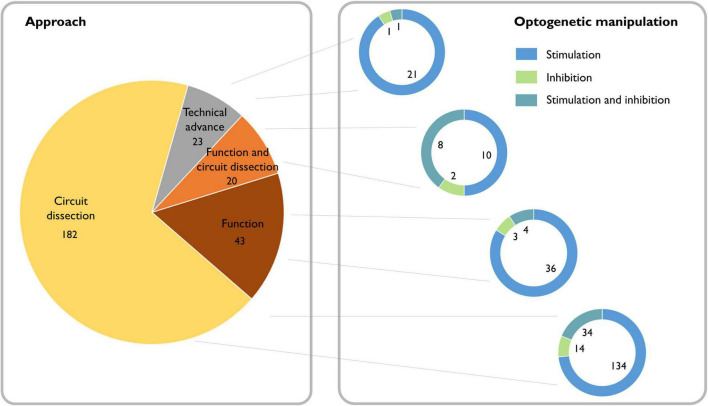
Literature review of the causal role of neuronal oscillations. Left: Searching PubMed with the terms “(oscillation OR oscillations) AND (optogenetics OR halorhodopsin OR archaerhodopsin OR channelrhodopsin)” from 2004 to the present yielded 377 papers. Of these, 49 did not address neural oscillations, 37 were review articles, 17 were computational/modeling-based, five did not make use of optogenetics, and one was a corrigendum. We classified the remaining 268 scientific articles containing experimental data based on whether the experiments addressed dissecting circuits, the function of oscillations, or neither (purely technical). Right: We further classified the papers based on whether they used optogenetic stimulation or inhibition and found that independent of the scientific question, optogenetic stimulation is the most broadly used technique.

Whether certain oscillations are required for behavioral output is a topic that still generates strong debate (Doelling and Assaneo, 2021). For many decades, lesion and pharmacological experiments in rodents, as well as observational studies of neurological and psychiatric patients, provided indirect evidence for the potential causal role of oscillations (Mitchell et al., 1982; Schnitzler and Gross, 2005; Haenschel et al., 2009; Mccarthy et al., 2011; Buzsáki and Moser, 2013). Now, with the emergence of optogenetic tools, we can inhibit or activate the neuronal population underlying a particular oscillatory activity with sufficient temporal precision to assess direct impact on behavior. Combined with electrophysiological measures of summed (LFP) and individual (single unit) neuronal activity, this approach provides an ideal framework to test whether these oscillatory dynamics are causally related to ongoing behavior, and whether functional accounts of specific rhythms hold up (see Box 2). A search of the literature reveals that since the introduction of optogenetic methods for manipulating neurons in 2005, 271 papers have been published applying this approach to investigating oscillations, which are split between 85% dissections of the neural circuitry underlying oscillations and 15% investigations of the function of oscillations (Figure 2).

To illustrate using optogenetics to link behaviorally relevant oscillations with the neural circuitry underlying them, we will describe the approach we recently took to unravel a slow oscillatory activity associated with stress in mice. The power of low-frequency rhythms increase in multiple brain regions during fear-inducing and stressful situations (Kumar et al., 2014; Karalis et al., 2016). We recently found that acute stress-induced low oscillations in the nucleus accumbens (NAc) predict subsequent deficits in reward seeking, and that NAc neurons whose firing rates decrease during the stressor are tightly synchronized with the oscillation, suggesting that the oscillation reflects a net inhibitory input to the area (Lowes et al., 2021). Subsequent coherence and directionality analyses demonstrate that the ventral tegmental area (VTA), a key component of reward processing circuitry, synchronizes at 2–7 Hz with the NAc during stress. Ultimately, optogenetic inhibition demonstrated the necessity of VTA activity for both the stress-induced oscillation and subsequent blunted reward seeking. Interestingly, this approach revealed that VTA inhibitory GABA neurons—and not dopaminergic neurons—decreased NAc stress-induced 2–7 Hz oscillatory power and restored reward-seeking behavior. This work provides an example of how optogenetic experiments can demonstrate the neural populations necessary for generating oscillations, and the impact of those neural populations on behavior. Demonstrating that the 2–7 Hz rhythm itself causes decreased reward-seeking behavior requires stimulation experiments (discussed below and in Box 2).

Notably, this example of stress-induced slow rhythms falls into the general category of supporting long-range network interactions. Slow oscillations have been suggested to synchronize distant areas (Jiang et al., 2017), and to organize the spiking of neurons across long-range circuits (Lisman and Idiart, 1995; Buzsáki, 2005; Benchenane et al., 2010; Reddy et al., 2021). Interestingly, in the case of VTA-NAc stress-induced oscillations, the synchronization reflects a net inhibitory influence. By contrast, low frequency oscillations in the low theta range are also observed in the prefrontal cortex (PFC) and ventral hippocampus (vHip) during anxiogenic conditions (Adhikari et al., 2010). In that situation, however, vHip theta oscillations do not affect the net firing rates within the PFC. Instead, optogenetic inhibition revealed that excitatory neurons from vHip that project to the PFC are necessary for theta synchrony, reorganizing PFC neuronal firing in order to modulate anxiety-like behavior (Padilla-Coreano et al., 2016).

These examples illustrate how optogenetic inhibition studies can identify the neural substrates of oscillatory activity underlying different aspects of behavior. These experiments also reveal the impact of the oscillation on local firing (e.g., modulating firing *rate* versus *timing*) as well as the specific neural subpopulation necessary for generating the oscillation. This approach has recently been exploited to show that different subpopulations of GABA interneurons in the primary visual cortex, such as parvalbumin- and somatostatin- containing neurons, are needed for spontaneous and visually induced gamma oscillations, respectively (Chen et al., 2017; Veit et al., 2017). In fact, optogenetic dissection of the neural basis of oscillations underlying behavior has become increasingly sophisticated, identifying the ion channels that confer rhythmicity (Ludwig et al., 1998; Varga et al., 2008; Vesuna et al., 2020), and impact specific behaviors (Vesuna et al., 2020).

While optogenetic inhibition is an ideal approach to determine the necessity of specific neurons and circuits for different behavioral outputs, it leaves open the question of whether the oscillations are sufficient to cause the behavior. Moreover, inhibition experiments often modulate multiple frequency bands at once, making it difficult to demonstrate that a particular rhythm is necessary for a given task. Along similar lines, it is difficult to disentangle whether behavioral changes result from decreased rhythmic activity *per se*, as opposed to a frequency-independent decrease of neuronal activity. For these reasons, optogenetic activation is a more appropriate technique to determine whether certain rhythms affect performance in a frequency-specific manner.

With optogenetic stimulation one can manipulate neuronal firing in a frequency-specific manner to create artificial oscillatory activity and evaluate the impact on behavior. For example, 4-Hz periodic stimulation of NAc-projecting VTA neurons was sufficient to recapitulate the low frequency NAc oscillations and subsequent reward seeking deficits observed in animals subjected to stress, whereas stimulating the same neurons at a faster rhythm (20 Hz) did not reproduce this phenotype (Lowes et al., 2021). Similarly, stimulating hippocampal terminals in the PFC with sinusoidal 8-Hz, but not 2- or 20-Hz, stimulation increased anxiety-like behavior (Padilla-Coreano et al., 2019). An important additional experiment to demonstrate that the rhythm causes the behavior would be to assess the effect of non-rhythmic stimulation of the same cells (see Box 2). These examples highlight the importance of recording the rhythmic circuit activity associated with a given behavior before using optogenetic manipulations to determine if that circuit is necessary for the behavior.

Nonetheless, optogenetic stimulation also has its limitations. Even when stimulating at a physiological frequency, it remains unclear whether optogenetic stimulation recapitulates the natural activity of neurons, or whether it elicits an artificial activity pattern that the neurons would otherwise never engage in. For example, while sinusoidal 8-Hz stimulation of vHip-PFC projections increased anxiety-like behavior, stimulating at the same frequency with pulses of light did not (Padilla-Coreano et al., 2019). Similarly, optogenetic activation of cells that encoded a shock-paired context within the dentate gyrus of the hippocampus elicited fear responses in a novel context, demonstrating a false memory recall (Ramirez et al., 2013). However, non-specific stimulation of the same area disrupts the fear memory encoding and recall (Kheirbek et al., 2013). When reconciling findings across optogenetic studies, it is important to consider technical aspects such as suboptimal expression of opsins or poor light penetration (see Yizhar et al., 2011 for review). However, it is likely that gain-of-function experiments can be best achieved by faithfully replicating the activity of circuits and neuronal downstream machinery of the naturally occurring oscillations. In fact, recent studies took advantage of this need for coordinated rhythmic activity across a long-range circuit to demonstrate that uncoordinated gamma-frequency stimulation disrupted behavioral performance (Fernández-Ruiz et al., 2021), whereas gamma synchrony between hemispheres is needed for tasks that require updating strategies (Cho et al., 2020). Crucially, optogenetic stimulation studies usually modify the firing rates of the stimulated neurons, thus failing to unequivocally prove that the generated rhythm rather than the altered firing is sufficient to modify behavior. While these two phenomena are difficult to disentangle, recent *in vitro* work has shown how distinct neuronal subtypes differently encode rate and rhythm information (Prince et al., 2021). Similar *in vivo* experiments assessing the effects of non-rhythmic, matched-rate, optogenetic stimulation on behavior can further help disentangle these factors (Box 2).

It is worth noting that oscillation-induced changes in behavior may not necessarily occur during the rhythmic activity. For example, we found that stress-induced oscillations led to changes in reward-seeking behavior for as long as 1 h after the activity had stopped (Lowes et al., 2021). This finding raises the intriguing possibility that some oscillations may represent network/circuit implementations of changes in synaptic plasticity, which are crucial for memory and learning (see Neves et al., 2008 for a review). Indeed, we recently showed that exposing mice to a novel environment induces a theta rhythm in the ventral hippocampus that weakens synaptic strength between the ventral hippocampus and medial prefrontal cortex, but facilitates both learning and optogenetic induction of long-term potentiation, a form of synaptic plasticity (Park et al., 2021). Interestingly, delta oscillations during sleep have been proposed as a homeostatic mechanism to rescale the potentiation in synaptic strength that occurs with prolonged wakefulness (Tononi and Cirelli, 2003). This mechanism is disrupted in patients with depressive disorders, possibly leading to the cognitive impairments seen in this population (Goldschmied and Gehrman, 2019). Optogenetic manipulations of delta rhythms during sleep and subsequent tests of synaptic plasticity (both *in vitro* and *in vivo*) provide exciting opportunities to test these hypotheses (Durkin et al., 2017).

Testing the hypothesis that slow wave oscillations during sleep modulate plasticity represents an example of using optogenetic manipulations to test the functional, rather than circuit-specific, role of oscillations. The application of optogenetics to understanding the functions of oscillations has lagged behind circuit-dissection approaches (see Figure 2) but has recently produced some exciting results. Of clinical interest, disruptions in gamma rhythms have been linked to cognitive disorders (Mably and Colgin, 2018) and gamma-frequency stimulation has been shown to improve cognitive impairments in genetic animal models of mental diseases (Cho et al., 2015, 2020; Iaccarino et al., 2016; Cao et al., 2018; Etter et al., 2019). From a more conceptual perspective, a recent study provided evidence that gamma coherence supports inter-area communication by showing that selectively perturbing gamma, but not theta oscillations, in different entorhinal cortical inputs to the hippocampus disrupts the information provided by the respective input (Fernández-Ruiz et al., 2021). Similarly, recent work demonstrated that simultaneous bilateral gamma stimulation, but not asynchronous or non-gamma frequency stimulation, increased gamma synchrony and restored cognitive flexibility (Cho et al., 2020). In contrast, another recent study used optogenetic manipulations and modeling to argue that long-range coherence simply reflects, but does not directly contribute to, communication between brain areas (Schneider et al., 2021). While these studies are unlikely to resolve this long-standing debate (Singer, 2018), they lay the groundwork for future experiments that interrogate the role of oscillations in circuit communication during behavior with optogenetic interventions. More broadly, these studies point the way for future research testing other hypotheses about the role of oscillations. For example, one could test the hypothesis that the alpha rhythm functionally inhibits irrelevant input by designing a task where one set of cues (e.g., visual stimuli) predict reward, while another set of cues (e.g., auditory stimuli) act as distractors. This experimental setup should show increased alpha oscillations in the distractor-cue sensory cortex, with corresponding decreases in firing rates. Crucially, optogenetically inhibiting the neural elements that generate alpha in the distractor-cue sensory cortex, or imposing a non-rhythmic firing pattern, would reveal its role in the task performance. Experiments using optogenetic manipulations during gamma oscillations have provided evidence that gamma oscillations enhance the processing of otherwise less salient stimuli (Siegle et al., 2014; Li et al., 2015; Ni et al., 2016). A similar approach can help resolve the functional role of beta oscillations during working memory, by revealing whether disrupting beta oscillations disinhibits the circuit or prevents the reactivation of information-encoding ensembles. A crucial consideration that is often neglected when using optogenetics to investigate the role of an oscillation in behavior, is that any intervention that changes the behavior may alter the oscillation indirectly (i.e., the optogenetic manipulation puts the animal into a new behavioral state that in turn impacts the oscillation). An important control for this confound is to conduct optogenetic manipulations that impact the oscillation without changing behavior (for example, unilateral inhibition in behavior that requires both hemispheres).

## Methodological Advances

As previously noted, human oscillation research has largely relied on M/EEG recordings, which may mostly reflect cortical activity. By contrast, invasive recordings in rodents have revealed subcortical rhythms. Moreover, cell-type specific optogenetic manipulations have demonstrated multiple overlapping sources for oscillations. While intracranial recordings have provided important insights regarding the relationship between different oscillatory frequencies and firing rates (Manning et al., 2009) or the influence of oscillations on single-neuron spike timing (Jacobs et al., 2007), there is a critical need for simultaneous EEG and intracranial recordings, preferably incorporating optogenetic interventions to help identify the circuits associated with oscillations observed extracranially. This data could in turn inform analytic efforts to improve source localization (Michel and Brunet, 2019; Seeber et al., 2019).

The recognition that oscillatory frequency can vary across subjects has also prompted advances in analytic methods. Historically, oscillations have been identified by decomposing the recorded signal into sinusoids of different frequencies and then computing their amplitude and frequency properties. Power tends to decrease with frequency (following a 1/f pattern), so to distinguish between true oscillations versus aperiodic changes in power, oscillations are typically identified as narrowband peaks in power above the non-oscillatory 1/f activity. However, changes in 1/f activity across development, species, or pathological conditions (Voytek et al., 2015; Peterson et al., 2017; Dave et al., 2018), frequency peak shifts with aging, clinical conditions, and GABA concentrations (Muthukumaraswamy et al., 2009; Haegens et al., 2014; Furman et al., 2018; Bódizs et al., 2021), changes in the rate of transient oscillatory events (Shin et al., 2017), or non-sinusoidal rhythms (Cole and Voytek, 2017), all can affect the estimation of oscillatory power. Ongoing efforts focused on improving current analytical methods for studying brain oscillations (Donoghue et al., 2020) show promise in developing estimates of neural activity frequency that account for individual differences.

Finally, as noted above, optogenetic stimulation can induce synaptic plasticity (Zhang and Oertner, 2007). Thus, an important consideration to take into account when incorporating optogenetic interventions into dissecting the function of oscillations, is that optogenetic interventions might themselves modulate circuit connectivity and impact behavior for this reason.

## Future Directions

In conclusion, optogenetic interventions show great promise in determining the functional consequence of oscillations. Here we suggest experimental advances to improve investigations of the necessity and sufficiency of rhythmic activity.

First, naturally occurring oscillations are often short-lived. Even when the pacemaker neurons that drive the rhythm are found, finding the temporal window(s) during which they set the pace and evoke a behavioral output can be difficult. “Closed-loop” optogenetic control of neurons based on ongoing activity was suggested several years ago (Grosenick et al., 2015), and has proven efficient at controlling seizures in rodents (Sorokin et al., 2017; Hristova et al., 2021). This approach could be used for manipulating neuronal activity selectively during relevant periods, such as when a specific oscillatory power exceeds or goes below a critical value (Van De Ven et al., 2016; Nicholson et al., 2018). This approach effectively impacts memory consolidation when applied to ongoing gamma oscillations in the amygdala region (Kanta et al., 2019), but studies on other frequencies are lacking. This technique is particularly suitable for addressing the role of oscillations, as it tightens the causal relationship between the neural activity and behavior-associated oscillations by minimizing non-specific stimulation.

Second, optogenetic studies comparing arrhythmic versus rhythmic stimulation patterns can provide useful information about whether increased neuronal activity is sufficient to impact behavior, or whether rhythmicity is required instead. This comparison would also be useful to assess how asynchronous activity of specific neurons impact broadband spectral power, and whether specific frequency bands are preferentially affected by changes in firing rate.

Third, as previously discussed, coherent oscillations between distant areas arise during different brain processes. A recent framework postulates that coherence mainly appears because spiking activity in the sending area causes post-synaptic potentials in other areas, reflecting anatomical connectivity (Schneider et al., 2021). Experiments examining the impact of synchronous vs. asynchronous optogenetic stimulation in two areas on neural coherence and task performance can provide evidence for the role of rhythmic activity in supporting inter-area communication, and the functional relevance of this connectivity. This approach has been successfully exploited to demonstrate the key role of prefrontal gamma coherence in rule updating tasks (Cho et al., 2020). Future studies using this approach with other task-relevant rhythms would advance the field.

Lastly, parallel studies comparing observations in rodents and humans are crucial for advancing the field. Specially, the combination of human non-invasive EEG or intracranial EEG, and invasive recordings with optogenetic manipulations in rodents can provide comprehensive information of relevant oscillations from the macro- to the micro-level, i.e., identifying the physiological properties, principal neurons, and proteins implicated in the generation of specific rhythms. A recently published study, which investigated the role of posteromedial 1–3 Hz rhythm in eliciting dissociative states (Vesuna et al., 2020), demonstrates with an unprecedented level of detail, the biophysical basis of a clinically relevant rhythm. Taking a similar approach to other brain oscillations will allow us to systematically resolve important outstanding questions such as how different oscillations are generated within different brain regions (Buzsaki and Wang, 2012; Colgin, 2013), how local is the LFP (Kajikawa and Schroeder, 2011; Herreras, 2016), and what function do oscillations play at the neurocomputational and behavioral levels. Doing so could additionally help disentangle rhythms implicated in pathological states, providing the basis for future treatments or new approaches to brain-related diseases.

## Author Contributions

II-L, SH, and AZH wrote the manuscript. SH and AZH provided funding. All authors contributed to the article and approved the submitted version.

## Conflict of Interest

The authors declare that the research was conducted in the absence of any commercial or financial relationships that could be construed as a potential conflict of interest.

## Publisher’s Note

All claims expressed in this article are solely those of the authors and do not necessarily represent those of their affiliated organizations, or those of the publisher, the editors and the reviewers. Any product that may be evaluated in this article, or claim that may be made by its manufacturer, is not guaranteed or endorsed by the publisher.
